# Candyflipping and Other Combinations: Identifying Drug–Drug Combinations from an Online Forum

**DOI:** 10.3389/fpsyt.2018.00135

**Published:** 2018-04-30

**Authors:** Michael Chary, David Yi, Alex F. Manini

**Affiliations:** ^1^Department of Emergency Medicine, NewYork-Presbyterian Queens, Flushing, NY, United States; ^2^Bronx High School of Science, Bronx, NY, United States; ^3^Division of Medical Toxicology, Department of Emergency Medicine, The Icahn School of Medicine at Mount Sinai, Flushing, NY, United States

**Keywords:** natural language processing, computational biology, computer simulation, psychedelic drug use, toxicology

## Abstract

Novel psychoactive substances (NPS) refer to synthetic compounds or derivatives of more widely known substances of abuse that have emerged over the last two decades. Case reports suggest that users combine substances to achieve desired psychotropic experiences while reducing dysphoria and unpleasant somatic effects. However, the pattern of combining NPS has not been studied on a large scale. Here, we show that posts discussing NPS describe combining nootropics with sedative-hypnotics and stimulants with plant hallucinogens or psychiatric medications. Discussions that mention sedative-hypnotics most commonly also mention hallucinogens and stimulants. We analyzed 20 years of publicly available posts from Lycaeum, an Internet forum dedicated to sharing information about psychoactive substance use. We used techniques from natural language processing and machine learning to identify NPS and correlate patterns of co-mentions of substances across posts. We found that conversations mentioning synthetic hallucinogens tended to divide into those mentioning hallucinogens derived from amphetamine and those derived from ergot. Conversations that mentioned synthetic hallucinogens tended not to mention plant hallucinogens. Conversations that mention bath salts commonly mention sedative-hypnotics or nootropics while more canonical stimulants are discussed with plant hallucinogens and psychiatric medications. All types of substances are frequently compared to MDMA, DMT, cocaine, or atropine when trying to describe their effects. Our results provide the largest analysis to date of online descriptions of patterns of polysubstance use and further demonstrate the utility of social media in learning about trends in substance use. We anticipate this work to lead to a more detailed analysis of the knowledge contained online about the patterns of usage and effects of novel psychoactive substances.

## 1 Introduction

Novel psychoactive substances (NPS) refer to novel synthetic compounds or derivatives of more widely known substances of abuse that have emerged over the last two decades (1). Examples include derivatives of cannabis, substituted phenylethylamines, or cathinones (bath salts). The term NPS may include substances used by other cultures, but new to Western users, such as khat (the progenitor of bath salts), kratom, or *Salvia*. The increasing use of NPS is linked with the rise of social media as a means to discuss NPS use and distribute the actual product (2).

The use of NPS is a public health concern. Use of substituted amphetamines is associated with sudden cardiac death and renal failure (3). Use of bath salts is associated with acute and persistent psychosis (3). Use of tryptamine derivatives is associated with psychosis and long-term psychiatric impairment, including anxiety and paranoia (4). The authors could find no study in the literature quantifying the impact of novel psychoactive substances in terms of disease-adjusted life years or monetary impact.

Chemical analyses of novel psychoactive substances voluntarily submitted by users suggest that novel psychoactive substances are frequently consumed with other substances rather than in isolation (5). A combination of substances may have fewer side effects than any individual substance. The term candyflipping refers to the combination of LSD and MDMA (Ecstasy) (6). This combination was first described in the early 1980s, a few years after MDMA became more widely available (7). Candyflipping seems to increase the potency and duration of MDMA-like effects, while decreasing the chance of overdosing on MDMA. MDMA is also known to be combined with other amphetamines, alcohol, and synthetic cannabinoids (8). Reports of polysubstance use may also reflect contamination during clandestine manufacture and dissemination.

Social media have emerged as informative sources of data for tracking behavior in the general population. Adolescents and young adults, the most widely described consumers of NPS (3, 9), frequently communicate candidly online. Whether the quality of data from social media allows is similar to that from more traditional means of syndromic surveillance is still being established. Credible doses of dextromethorphan can be inferred from YouTube comments (10). Estimates of the geographic distribution of opioid misuse across the United States from Twitter have outstanding agreement with those from the National Survey on Drug Usage and Health (11). Language on Twitter correlates with the geographic distribution of heart disease (12).

Traditional means of syndromic surveillance are difficult to apply to the epidemiology of novel psychoactive substances. National surveys, such as the National Survey on Drug Usage and Health, occur once a year and involve in-person interviews. Analyses of calls to poison control centers or encounters with health care providers provide a biased picture of the patterns of NPS usage.

Our approach had two broad aims:

1. Demonstrate that data concerning polysubstance use could be extracted from online user posts
2. Demonstrate that from these data we could infer novel as well as known combinations of substances.

Inferring known combinations of substances will bolster the credibility of online posts as a source of this type of data. Our approach was to use techniques from natural language processing and Big Data to analyze Lycaeum. Lycaeum is a website and Internet forum dedicated to promoting information about psychoactive substances (13).

## 2 Materials and Methods

### 2.1 Overview

We wrote software in the programming language Python (14) to extract user posts from Lycaeum, identify novel psychoactive substances, and analyze the content of the posts. Posts consist of unstructured text, also called freetext, similar to the “Comments” section after online articles in the New York Times or Financial Times web sites. We included only public posts for analysis. We omitted posts that were marked as deleted or flagged by the moderator.

### 2.2 Acquisition of User Posts

We developed a web scraper with the Python package *scrapy* (15) to extract all accessible posts (*n* = 9,289) from the start of Lycaeum in 1996 until December 2016. We lemmatized the posts and removed stopwords using *nltk*, the Python Natural Language Toolkit (16). Lemmatization refers to the conversion of all lexical and semantic variants of a word to one base form. One lemmatizes, for example, *reading, reads*, and *reader* to read. Lemmatization is one way to move from the actual unstructured text to a tractable representation of the underlying semantics. Removing stopwords refers to filtering out words such as “the,” or “a,” which occur often but add little information to the text. Removing stopwords is a common approach to make the frequency of words more accurately approximate the relative prevalence of concepts in a piece of text.

### 2.3 Identification of Substances

We used a three-step process to identify substances. We used *nltk* to identify all nouns before lemmatization. Authors MC and AM each individually manually curated this list to identify those nouns that likely referred only to drugs. Only nouns that were identified by both AM and MC as likely relating only to drugs were used for subsequent analysis. Author DY cross-referencing this list with Wikipedia, PubChem, and DrugBank to provide the standard spelling and a list of synonyms for each potential substance. This cross-referencing, for example, mapped *xanny*, a variant of Xanax to alprazolam. Authors DY and MC annotated each drug as to its drug class. We considered the following classes: sedative-hypnotic, hallucinogen, stimulant, nootropic, psychiatric, anticholinergic, analgesic, antipyretic, antiemetic, antihypertensive, cannabinoid, or contaminant.

### 2.4 Calculation of Correlation

To identify patterns of co-mentions of substances, we created a *drug-post matrix*, such that the *ij*th entry of this matrix is 1 if drug *i* is mentioned in post *j* otherwise −1. We then calculated the correlation between the patterns of mention of all pairs of drugs across the Lycaeum corpus. We calculated the correlation between any two drugs, *a* and *b*, as the inner product of the corresponding rows in the drug-post matrix, normalized by the number of posts *n*, $r_{a,b}=\vec{a}\cdot \vec{b}/n$. Expressed another way, we treated each drug as a multidimensional vector. Each dimension corresponds to a post. The correlation between any two drugs over posts is the cosine of the angle formed between the two corresponding vectors. The equation presented before calculates that angle’s cosine. This equation is adapted from Ref. (17). We obtained a threshold for statistical significance for the correlation between drug *a* and drug *b, r_a_,_b_*, by randomly shuffling the drug-post matrix 10,000 times and recalculating all drug pair correlations to derive an empiric probability distribution function for *r_a_,_b_*.

## 3 Results

**Figure 1:**
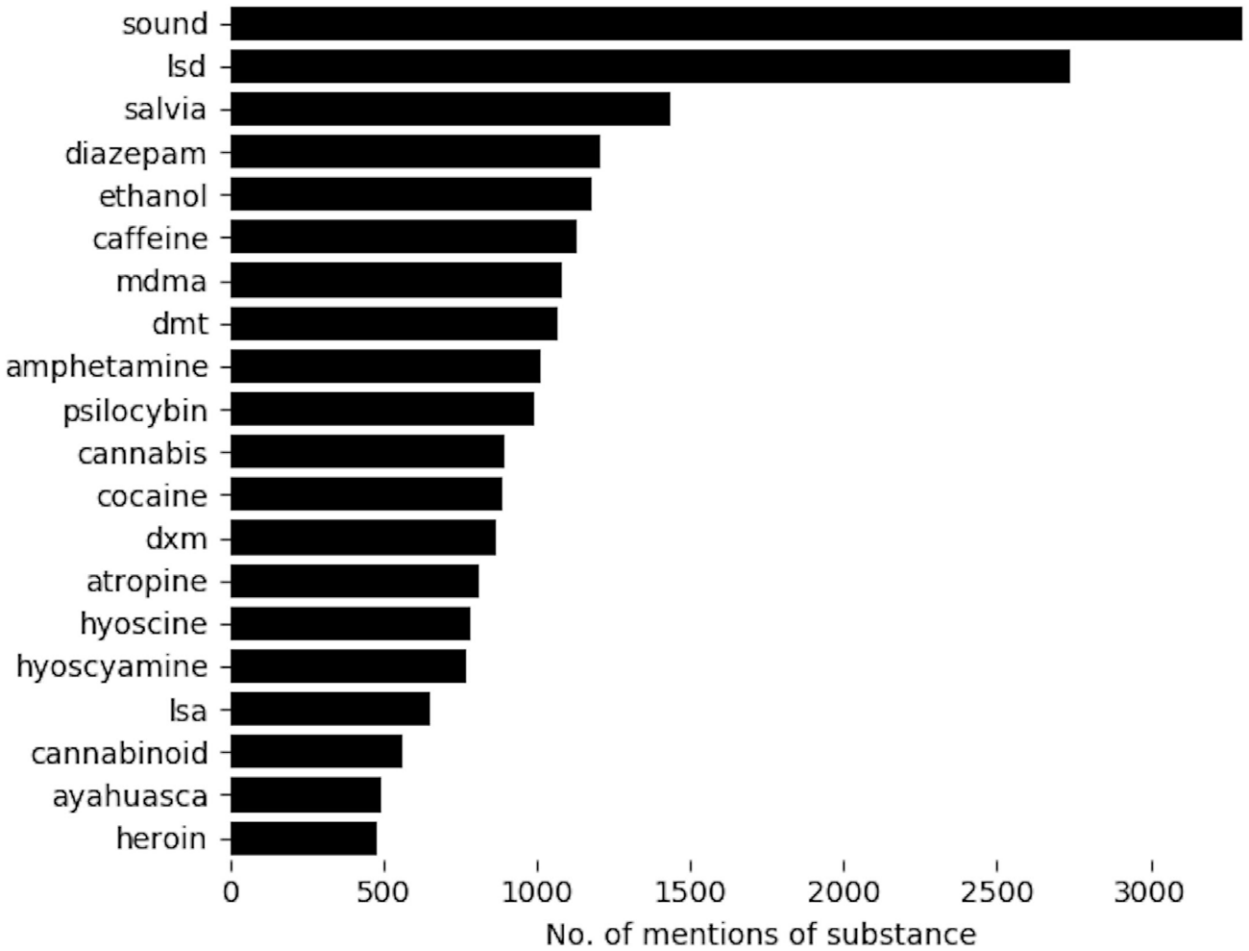
Top 20 most frequently mentioned substances. *x*-Axis denotes number of posts in which the substance was mentioned at least once. MDMA, 3,4-methylenedioxymethamphetamine, also known as ecstasy; DMT, *N,N*-dimethyltryptamine; DXM, dextromethorphan; LSA, lysergic acid amide, also known as ergine.

The 20 most frequently mentioned substances included common hallucinogens, stimulants, sedative-hypnotics, as well as, interestingly, sound (Figure 1). The *x*-axis in Figure 1 shows the number of posts that mention the substance at least once. In the following paragraphs, we discuss some of these substances in detail as they may be unfamiliar to the reader.

We amalgamated the phrases *binaural beats, binaural sound*, and *binaural music* onto the token *sound*. All these refer to the presentation to each ear of pure-tone sine waves differing only by frequency. Posts to Lycaeum frequently described listening to binaural beats while using substances to enhance the experience. Binaural sound may enhance concentration on a task when compared to silence (18). It has not been demonstrated to alter emotional arousal (19). The authors could find no study investigating the combination of binaural sound with any psychoactive substance, despite its prevalence in our data set. We excluded mentions of binaural beats from subsequent analyses as the focus of this study was on drug–drug combinations. It is unclear why posts mentioned these sounds so frequently. A detailed analysis of the context in which *binaural beats* were mentioned was beyond the scope of this study.

LSD (lysergic diethyl amide) is a canonical hallucinogen (18). Salvia, i.e., *Salvia divinorum*, refers to a psychoactive plant from Oaxaca, Mexico rich in salivinorin A, a *κ* opioid receptor agonist (20).

Diazepam is a benzodiazepine sedative-hypnotic sold in the US under the trade name Valium. Ingesting diazepam along with a hallucinogen may mitigate the anxiety, dysphoria, or rapid heart rate associated with some hallucinogens. Co-ingestion of a sedative-hypnotic and hallucinogen may potentiate hallucinogen’s intended effect (21). The administration of benzodiazepines is part of the initial treatment of symptomatic overdoses of hallucinogens (22). Ethanol and caffeine are widely consumed psychoactive substances. MDMA (3,4-methylenedioxymethamphetamine; also called ecstasy) is the canonical entactogen–empathogen, a substance that enhances feelings of closeness, bondedness, empathy, and sexual attraction (23). DMT (*N,N*-dimethyltryptamine) is a hallucinogenic derivative of tryptamine. It is considered the main psychoactive compound in hallucinogenic plants such as *Mimosa tenuiflora* (24) and the melange ayahuasca (25). Amphetamine (also called *speed*) is a long-recognized stimulant. Psylocybin is another canonical hallucinogen; it is the active ingredient in “magic mushrooms” (26).

Atropine, hyoscine (also called scopolamine), and hyoscyamine are components of jimson weed, a soporific and hallucinogen. LSA (lysergic acid amide; also called ergine) is an ergot related to LSD and the most investigated hallucinogen in morning glory (27). It emerged as an alternative to LSD; popular articles suggest that LSA is also a point of comparison when describing the effects of methylone (28).

Cannabis is a commonly consumed sedative, although some strains may have hallucinogenic or stimulatory effects (29). The term *cannabinoid* likely refers to synthetic cannabinoids. Synthetic cannabinoids are agonists at cannabinoid receptors as well as dopaminergic, sertoninergic, and adrenergic receptors; synthetic cannabinoids may be more likely to precipitate psychosis than cannabis (30).

**Figure 2:**
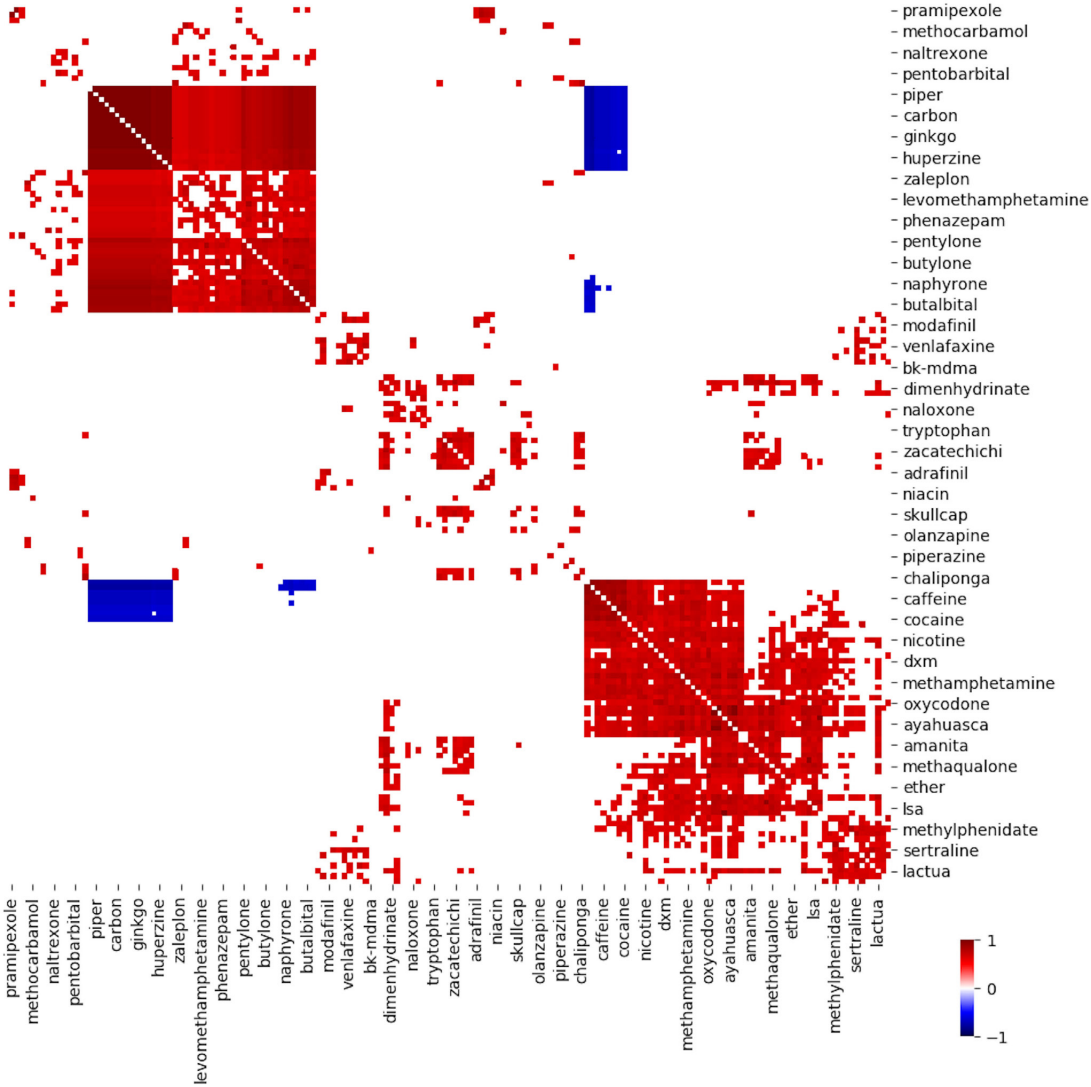
Heat map of correlation coefficient of substance–substance co-mention pairs whose correlation was statistically significant. Each tiny box represents one pair of substances. Drug names on *x*- and *y*-axis specify the pair associated with each box. Color of tiny box indicates correlation, according to scale up in lower right.

To better understand how posts described combining substances, we calculated the correlation across all documents for all pairs of substances. Figure 2 shows all combinations whose correlations are statistically significant. We used bootstrapping (see Materials and Methods) to determine the threshold for statistically significant correlations.

Figure 2 is a clustered heat map, a graphical depiction of the *drug-post* matrix. The color of the *ij*th box indicates the correlation between drug *i* and drug *j*. Warmer colors indicate correlations closer to 1. Colder colors indicate correlations closer to −1. This heat map is symmetric across the diagonal because the correlation between drug *i* and drug *j* is the same as the correlation between drug *j* and drug *i*. The diagonal is not drawn to avoid a ceiling effect distorting the figure. The orders of substances on the *x* and *y* axes are the same. The ordering of substances along the *x*-axis is the same as that along the *y*-axis. This ordering was chosen to group together pairs of drugs with similar correlations.

Three large clusters are apparent. Proceeding from left to right along the horizontal axis, one cluster begins with pramipexole and ends with butalbital. This cluster contains substances commonly labeled as nootropics (pramipexole, ginko, levomethamphetamine) or cathinones (bath salts; pentylone, butyrone, naphyrone). The next cluster begins with modafinil and ends with chaliponga. It contains hallucinogenic plants (zacatechichi, chaliponga) and psychiatric medications (venlafaxine, olanzipine). The third cluster contains stimulants (caffeine, cocaine, nicotine, methylphenidate) and hallucinogenic plants. The mostly blue square in the lower left indicates that compounds from the first cluster (nootropics and cathinones) are rarely discussed with compounds from the third cluster (stimulants and certain hallucinogenic plants). A negative correlation (blue color) between two substances means that when the first substance is mentioned the second substance is less likely to be mentioned. It does not mean that when one substance is mentioned posts explicitly discuss avoiding the second substance.

The term piper likely refers to *Piper methysticum* a source of kava, a herbal anxiolytic (31). Piper may also refer to phenylpiperazines, a novel class of stimulants marketed as alternatives to bath salts (32). Huperzine is an acetylcholinesterase inhibitor marketed as a nootropic (cognitive enhancer), although human studies show minimal effects (33).

Figure 2 demonstrates the face validity of this approach to toxicosurveillance and provides novel insights. Caffeine is a common adulterant in samples of cocaine (34, 35). Those who use cocaine are more likely to consume nicotine and caffeine (36).

The correlation between patterns of mention of pentylone, butylone, and naphyrone (upper left group) likely reflects debates on the relative effects of each substance, although they might reflect unreported patterns of use. A novel finding is that discussions mentioning bk-MDMA (also called methylone), another cathinone, significantly frequently mentioned methamphetamine, and hallucinogens (bufotenin, mimosa), but not other bath salts. Amphetamines are a frequent contaminant of bath salts (37).

Some reported patterns of use are not observed here. Figure 2 shows no significant co-mentions of monoamine oxidase inhibitors (MAOIs) with derivatives of tryptamine, such as dimethyltryptamine. Monoamine oxidase inhibitors (MAOIs) potentiate dimethyltryptamine by preventing metabolism of DMT in the gastrointestinal tract (25). *Mimosa* (38) and *chaliponga* (39) are plant sources of DMT. Ayahuasca is a source of DMT used in South American religious ceremonies and increasingly used in the United States (40). Harmaline is a *β*-carboline RIMA (reversible inhibitor of monoamine oxidase A (41)). Perhaps because the combination of MAOIs and hallucinogens has been described (42), the topic is assumed knowledge in online fora. Or, the topic may be more discussed in other fora.

**Figure 3:**
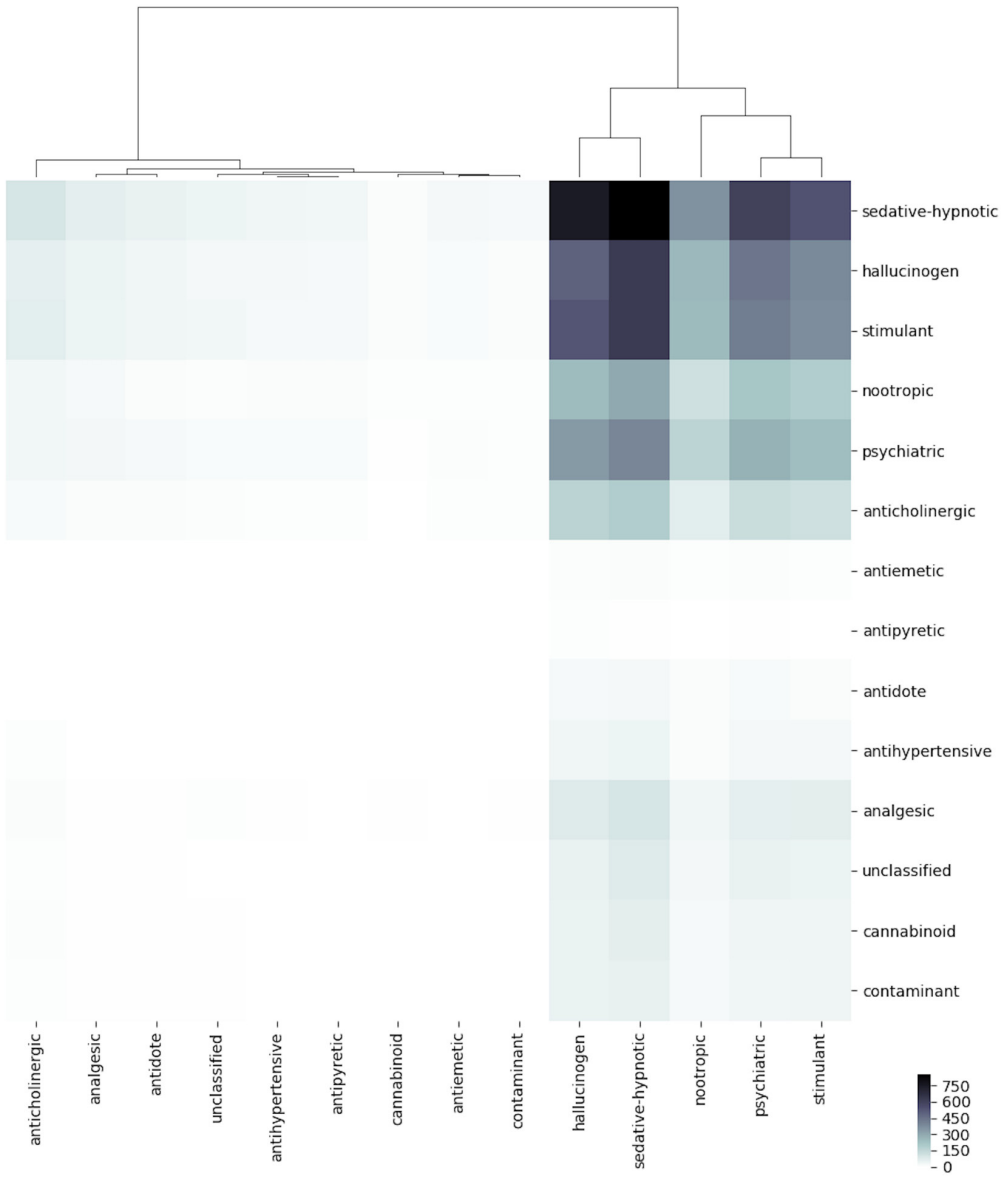
Heat map of substance-substance co-mentions by class. Each tiny box represents one pair of substance classes. Labels on the *x*- and *y*-axes specify the substance classes associated with each box. Color of tiny box indicates absolute frequency of mentions, according to colorbar scale in lower right.

To identify patterns of co-ingestion across classes, Figure 3 groups substance mentions by class. The most commonly co-mentioned classes are sedative-hypnotics, hallucinogens, and stimulants, followed by nootropics, psychiatric medications, and anticholinergics. For the purpose of Figure 3, each drug was assigned to one class only. In reality, a drug may have multiple effects, with only different effects manifesting at various doses. We chose the class that reflects the effects of the drugs at commonly ingested doses. We, for example, would classify diphenhydramine (Benadryl) as an antihistamine even though it is an anticholinergic at higher doses. We were unable to extract dosing information to further guide classification.

**Figure 4:**
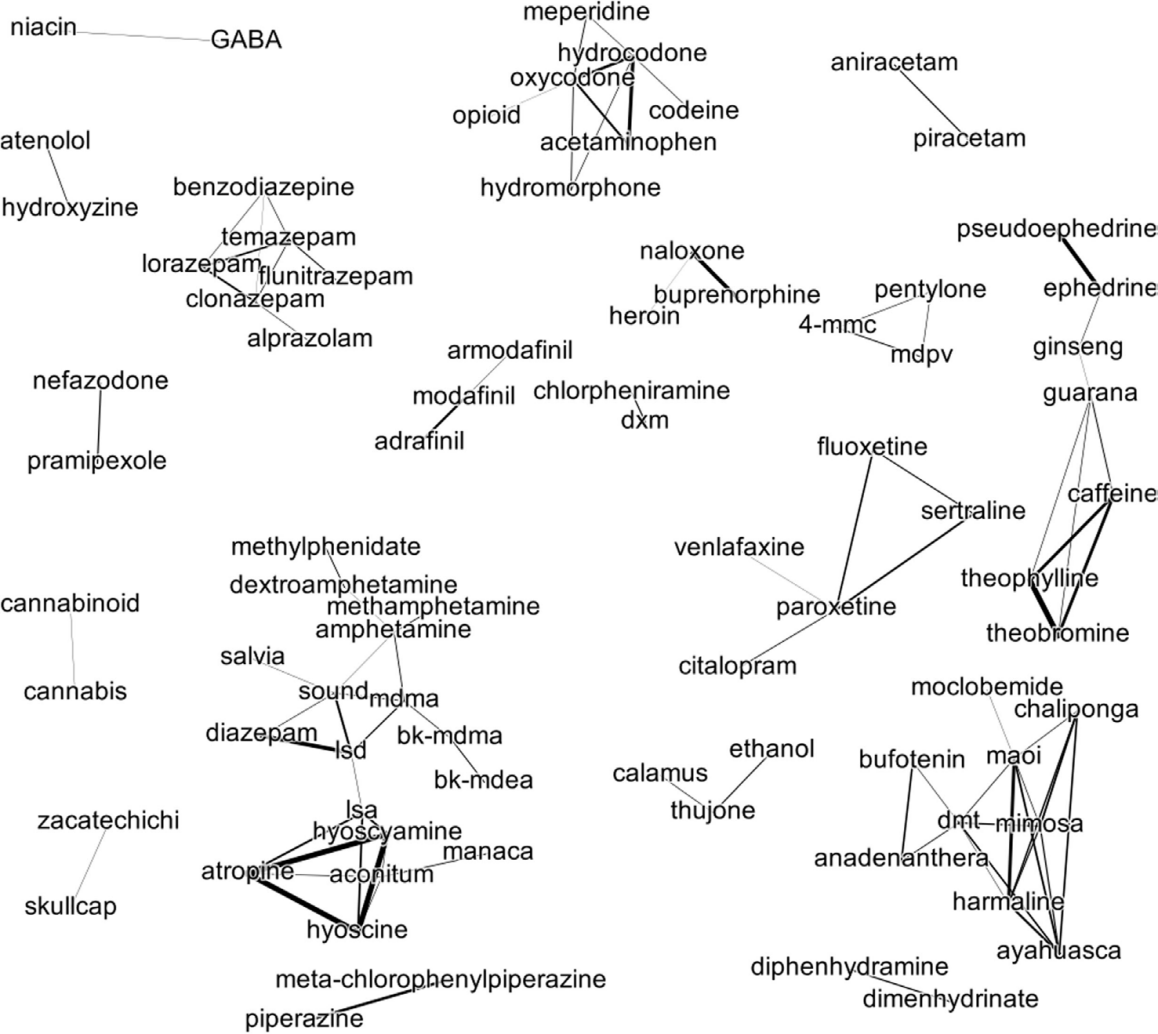
Social network of drug discussions. Each node (text) represents a substance. Each edge (connecting line) represents the correlation between mentions of the two connected substances. The thicker the line the stronger the correlation.

To identify patterns of substance use involving more than two substances, we constructed a social network of drugs (Figure 4) as follows. We created a connection between two drugs if those two drugs had a significant correlation. We depicted that connection graphically as a line. The width of the line reflects the strength of the correlation. Piecing together these pairwise connections creates a larger network as follows. Drug A develops an indirect connection to Drug C through Drug B if the patterns of mention of Drug A and Drug B are correlated as well as the patterns of mention of Drug B and Drug C.

We identified six groups containing more than three members. We term these larger groups semantic islands. Posts that mention one drug in a semantic island usually only mention substances from that same island if they mention more than one substance. There is an opioid island in the center top. Proceeding clockwise there is a stimulant island (caffeine is the hub), an SSRI hub (paroxetine is the hub), a plant hallucinogen island (DMT and mimosa are the hubs), a synthetic hallucinogen island (LSD and sound are the hubs), and a benzodiazepine island.

The SSRI island is formed by citalopram, sertraline, paroxetine, fluoxetine, and venlafaxine. In the SSRI island, paroxetine forms the hub it is directly connected to every other member of the island. One interpretation of this arrangement is that paroxetine (trade name Paxil) forms a frame of reference for evaluating other SSRIs.

In the synthetic hallucinogen, LSD is a hub that bridges two subislands. The left subisland of the hallucinogen island contains substances canonically thought to be anticholinergic. Hyoscine and hyoscyamine are tropane alkaloids found in jimson weed. The right subisland contains amphetamine derivatives, such as MDMA and the MDMA derivatives (bath salts), bk-MDMA (*β*-keto MDMA; methylone) and bk-MDEA (ethylone).

The triad formed by ethanol, calamus, and thujone reflects discussion on absinthe, which was thought to have hallucinogenic properties. Aging alcohol in wormwood was thought to infuse the solution with *α*-thujone. Calamus, referring to *Acorus calamus*, was also thought to be a hallucinogenic component of absinthe.

The triad formed by armodafanil, modafinil, and adrafinil reflect discussions on how to obtain modafinil without a prescription. Modafinil (trade name Provigil) and Armodafinil (trade name Nuvigil) are currently only available with a prescription in the United States. Adrafinil is metabolized to modafinil and is not designated a controlled substance in the United States.

The connection between niacin and GABA refers to anecdotal reports that combined oral administration of niacin and GABA increases the amount of GABA that crosses the blood–brain barrier. To the authors’ knowledge, there are no peer-reviewed reports on this. Nor have there been reports of combining pramipexole (a dopamine agonist) with nefazodone (an SSRI).

## 4 Discussion

This study presents the first formal analysis of patterns of discussion in online fora describing patterns of substance–substance co-ingestion. Our aim was to simultaneously demonstrate the validity of using internet fora for syndromic surveillance and discover novel substance–substance co-mentions. Our analysis of Lycaeum identified 183 combinations. Of those combinations, 44 have never been directly studied but are similar to combinations that have been directly studied. Three combinations, nefazodone and pramipexole, zacatechichi (mugwort) and skullcap, and niacin and GABA, have no antecedents in the literature.

We found that conversations mentioning synthetic hallucinogens tended to divide into those mentioning hallucinogens derived from amphetamine and those derived from ergot. Conversations that mentioned synthetic hallucinogens tended not to mention plant hallucinogens.

We also found that bath salts are commonly discussed with sedative-hypnotics and nootropics, while more canonical stimulants are discussed with plant hallucinogens and psychiatric medications. Discussions that mention sedative-hypnotics most commonly also mention hallucinogens and stimulants. Substances across all classes are frequently compared to MDMA, DMT, cocaine, and atropine when trying to describe their effects.

There are many limitations to this study. It analyzes the patterns of discussion of those who chose to share information about patterns of drug use. There are no analytic data to support that any substances mentioned together were ingested together. This study did not perform a detailed linguistic analysis of all text. A “co-mention” between drug *i* and drug *j* could mean ingesting drug *i* and drug *j*, avoiding the co-ingestion both substances, or consuming one but not the other. We looked for explicit mentions of each substance.

It is possible that posts mask mentions of usage with slang, even in online fora dedicated to discussion about novel psychoactive substances. To the knowledge of the authors, there exists no comprehensive or independently validated dictionary of slang relating novel psychoactive substances. We attempted to standardize vocabulary using manual curation. The classification system used in Figure 2 deviates from accepted best practices in biomedical ontology. For example, *anticholinergic* and *contaminant* are not mutually exclusive and describe properties at different levels of abstraction. The former term describes a binding property of the chemical. The latter term describes a property a substance has by virtue of its location. The term *citalopram* is not a property but a substance. The classification system also simplifies the reality that many NPS bind to many receptors and have active metabolites. We chose this simple classification system to reflect the categorization used by clinicians. Subsequent investigations that aim to join data from social media with existing knowledge repositories may have to develop a more formal and logically consistent representation of knowledge in this domain.

The textual analysis is also limited in that no attempt was made to infer why posts selected one pair of substances over another. Perhaps more sophisticated techniques from natural language processing or artificial intelligence could uncovert such latent variables.

## Author Contributions

MC wrote the software to analyze the data from Lycaeum, manually curated some drug categories, wrote, and edited the manuscript. DY wrote the software to acquire the data from Lycaeum and manually curated some drug categories. AM provided guidance during analyzing the data and helped revise the manuscript.

## Conflict of Interest Statement

The authors declare that the research was conducted in the absence of any commercial or financial relationships that could be construed as a potential conflict of interest.
